# Prediction of Radiation Treatment Response for Locally Advanced Rectal Cancer via a Longitudinal Trend Analysis Framework on Cone-Beam CT

**DOI:** 10.3390/cancers15215142

**Published:** 2023-10-25

**Authors:** Zirong Li, Ann C. Raldow, Joanne B. Weidhaas, Qichao Zhou, X. Sharon Qi

**Affiliations:** 1 Manteia Medical Technologies Co., Milwaukee, WI 53226, USA; lizirong@manteiatech.com; 2 Department of Radiation Oncology, University of California Los Angeles, Los Angeles, CA 90095, USA; araldow@mednet.ucla.edu (A.C.R.); jweidhaas@mednet.ucla.edu (J.B.W.)

**Keywords:** locally advanced rectal cancer, cone-beam computed tomography, longitudinal trending analysis, radiomics

## Abstract

**Simple Summary:**

Locally advanced rectal cancer (LARC) is commonly treated with neoadjuvant chemoradiation (CRT) followed by total mesorectal excision (TME). Patients respond to the CRT differently due to inter- and intra- patient variability in radiosensitivity. 15–27% of patients completely respond to the CRT and achieve pathologic complete response (pCR), while a large proportion of patients have a partial or poor response. TME is a highly invasive procedure, which can cause overtreatment, leading to morbidity and even mortality for the LARC patients who had a pCR. This work introduces an integrated framework to inform clinical decision making through systematical analysis of longitudinal Cone Beam Computed Tomography (CBCT) during a treatment course. The effectiveness and robustness of the response prediction model, based on quantitative imaging features derived from targeted volume from pre- and during-treatment images, were validated on a retrospective cohort of LARC patients towards personalized treatment.

**Abstract:**

Locally advanced rectal cancer (LARC) presents a significant challenge in terms of treatment management, particularly with regards to identifying patients who are likely to respond to radiation therapy (RT) at an individualized level. Patients respond to the same radiation treatment course differently due to inter- and intra-patient variability in radiosensitivity. In-room volumetric cone-beam computed tomography (CBCT) is widely used to ensure proper alignment, but also allows us to assess tumor response during the treatment course. In this work, we proposed a longitudinal radiomic trend (LRT) framework for accurate and robust treatment response assessment using daily CBCT scans for early detection of patient response. The LRT framework consists of four modules: (1) Automated registration and evaluation of CBCT scans to planning CT; (2) Feature extraction and normalization; (3) Longitudinal trending analyses; and (4) Feature reduction and model creation. The effectiveness of the framework was validated via leave-one-out cross-validation (LOOCV), using a total of 840 CBCT scans for a retrospective cohort of LARC patients. The trending model demonstrates significant differences between the responder vs. non-responder groups with an Area Under the Curve (AUC) of 0.98, which allows for systematic monitoring and early prediction of patient response during the RT treatment course for potential adaptive management.

## 1. Introduction

Colorectal cancer is the fourth most common cancer diagnosed in the United States and has the second-highest mortality rate among all cancer types [1]. Locally advanced rectal cancer (LARC) is commonly treated with neoadjuvant chemoradiation (CRT) followed by total mesorectal excision (TME) and adjuvant chemotherapy [2,3]. While all patients with the locally advanced disease receive neoadjuvant radiation, not all patients benefit equally. Approximately 15–27% [4,5] of patients completely respond to the neoadjuvant CRT and achieve a pathologic complete response (pCR), while a large proportion of patients have a partial or poor response. Given that TME is a highly invasive procedure, the current one-size-fits-all approach can cause overtreatment [6,7,8,9], leading to morbidity and even mortality for those LARC patients who had a pCR. For non-responders, neoadjuvant CRT may not provide enough benefit, and alternative or additional strategies may be indicated.

A major challenge for LARC management is distinguishing patients who may respond to RT from those who may not on an individual level. In recent years, advanced cross-sectional and functional imaging enables non-invasive characterization of the tumor, making it possible to establish pre-treatment biomarkers for the accurate prediction or early assessment of post-RT response to improve prognostication and patient stratification towards an optimal outcome. Indeed, comprehensive cancer characterization based on deep learning (such as tumor heterogeneity) derived from medical imaging could improve patient risk classification and optimize management [10,11,12]. Quantitative imaging features have been widely utilized to extract patient-specific information from medical images to predict the effectiveness of radiotherapy [13,14]. More and more research has shown the potential predictive power of CT-based radiomic signatures in identifying patients with postoperative pathological good responses [15,16,17,18].

In-room volumetric cone-beam computed tomography (CBCT) is widely used to ensure proper alignment but also allows us to assess tumor response during the entire treatment course. The CBCT-based radiomic provides a large number of potential features, as well as feature changes in those features, thus offering additional predictive values of tumor response [19,20,21,22]. Many researchers have begun to pay attention to daily images during treatment, and many publications have proved the values of delta-radiomic research during treatment [23,24,25,26,27]. Plautz et al. demonstrated that changes in features extracted from daily images could be caused by treatment and proved their potential in the early assessment of treatment response [28]. Nasief et al. proposed a prognosis analysis based on delta-radiomic features for a cohort of pancreas patients (AUC = 0.94) [29]. Boldrini et al. demonstrated that delta-radiomic and imaging features variation during magnetic resonance-guided radiotherapy treatment may represent a cCR prediction tool in locally advanced rectal cancer [30].

Traverso et al. reviewed 41 literatures and evaluated the methodological details in each study, they concluded that the repeatability and reproducibility of radiomic features are sensitive to image quality and software used to extract radiomic features. Only a few features are reproducible, some features seem impossible to be reproducible, which makes it difficult to replicate others’ conclusions [31]. Delta-radiomic features, involving feature variations at different acquisition time points, offer additional potentials and benefits for experimental reproducibility. Nardone et al. demonstrated that delta-radiomic features are more robust than traditional radiomic features [32]. The feasibility of CBCT radiomic features largely depends on the quality of the radiomic features. Delgadillo et al. conducted research on the repeatability and reproducibility of CBCT, quantifying repeatability by correlating the radiomic features obtained from 20 repeat CBCT scans within 15 min. However, these radiomic features were reproducible in more than the 9.8% of reconstruction and preprocessing methods [33]. Despite of great potentials, the utilization of a longitudinal CBCT-based radiomic analysis for early response prediction has not been widely adopted at clinic possibly due to (1) tedious steps requiring additional resources and manpower, and (2) the lack of an integrated framework that truly benefits clinical decision-making. 

In this work, we proposed a longitudinal radiomic trend (LRT) framework to monitor and forecast potential treatment response based on series of image features derived from daily CBCT during a treatment course. To this end, we developed an automated platform to facilitate clinical implementation. The LRT framework consists of four modules: (1) Automated registration of CBCT scans to planning CT and evaluation; (2) Feature extraction and normalization; (3) Longitudinal trending analyses; (4) Feature reduction and model creation. The proposed framework allows for more robust treatment response assessment through daily CBCT analyses for personalized medical advice.

## 2. Materials and Methods

### 2.1. Overview of Longitudinal Radiomic Trend (LRT) Framework

**Figure 1:**
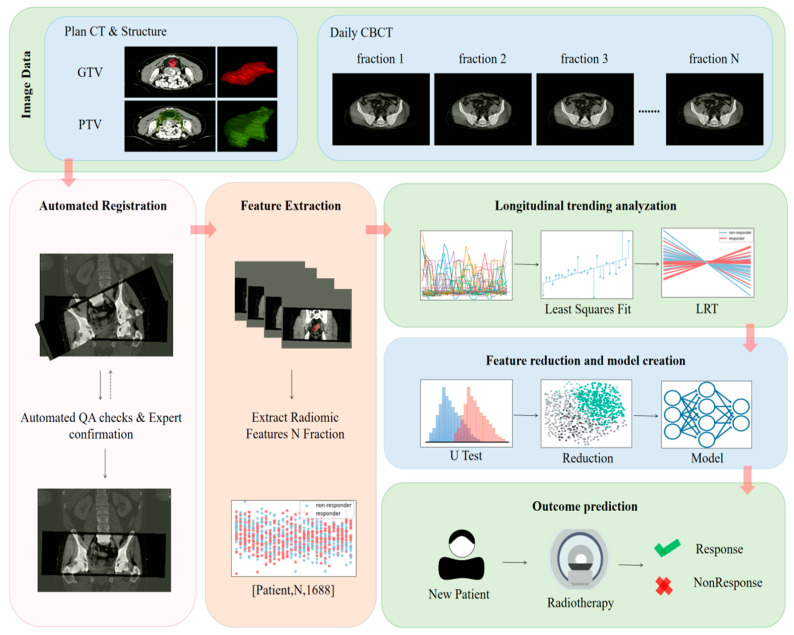
The schematic workflow of longitudinal radiomic trend framework (LRT).

We developed a framework to facilitate clinical decision-making that involves daily CBCT images during the entire treatment course, alongside the localization CT image and initial plan contour during treatment. Figure 1 shows the schematic process for the proposed framework. The process and all the details are explained further below.

#### 2.1.1. Automatic Registration and Evaluation

Daily pre-treatment CBCT images are subjected to rigid registration with the planning CT images. In our framework, we used rigid transformation based on mutual information and automatically evaluated the consistency of the displacement matrix of each daily CBCT registration for each patient. Unexpected registration results were re-checked and registered manually. The automatic consistency assessment is based on manually checking the accuracy of the registration of the first day’s images and then comparing the subsequent registration deformation matrix with the first day’s deformation matrix. If the difference is higher than 5%, a warning is issued requiring further manual verification. Finally, all registration results were reviewed to guarantee the reliability of the registration results.

#### 2.1.2. Feature Extraction and Normalization

The gross tumor volume (GTV) and planning tumor volume (PTV) were delineated on planning CT, with guidance of MRI and/or PET for treatment planning purposes. The original GTV and PTV contours were then transferred to the fused CBCT images, from which radiomic features were extracted separately for the GTV and PTV. While shape features were extracted from the original image without transformation, resulting in a total of 14 features. The images underwent 7 transformations for other feature extractions. These transformations include Laplacian of Gaussian, Wavelet, Gradient, SquareRoot, Logarithm, Exponential, and Square. Among them, Laplacian of Gaussian was performed 4 times with sigma values of 0.6, 1.0, 3.0, and 5.0. Wavelet was combined with high-pass and low-pass filters 8 times, resulting in 18 types of images including the original image. Each transformed image contained 93 features, including 18 first-order features, 24 GLCM features, 16 GLRLM features, 16 GLSZM features, 14 GLDM features, and 5 NGTDM features. For patients who received N fractional treatments, N groups of feature sets were calculated based on the GTV area and PTV from the daily CBCT, resulting in a total of 1688 imaging features in each group. Feature extraction was performed using PyRadiomics (version 3.0.1) [34]. For each patient, we investigated the trend of each feature’s changes, rather than focusing on the magnitude of the values. To this end, we performed Z-score tests using each patient’s daily feature mean and variance to standardize, and the absolute value of 3 was imposed as the outlier boundary [35].

#### 2.1.3. Longitudinal Radiomic Feature Trending 

Quantitative image features, captured by daily CBCT scans, can be useful to assess the change of radiomic features in a longitudinal pattern, which, in turn, provides early prediction to identify, quantify, and potentially predict therapy-induced changes over the course of treatment. For each fraction of *j* (*j* = 1, 2, …, N) of each patient, the feature (*i* = 1, 2, …, 1688) extracted from each CBCT scan, let $X_{j}$ denote the number of delivered fractions.

The least-squares method [36,37] was employed to fit the features over the course of treatment, focusing on the trending of feature changes to avoid atypical features and noise in the images. For each patient, assuming a linear relationship. Equation (1) is used to capture the correlation between each radiomic feature $RF_{ij}$ and the number of fractions $X_{j}$.

(1) $$RF_{ij}=LRT_{i}\cdot X_{j}+\beta_{i}$$

where the slope $LRT_{i}$ can be calculated in Equation (2), and the intercept of the fitted feature line $\beta_{i}$ can be calculated by Equation (3). Calculate the fitting error for each feature by Equation (4).

(2) $$LRT_{i}=\frac{\sum_{j}^{n}X_{j}\cdot RF_{ij}-n\overset{¯}{X}\cdot \overset{¯}{RF_{i}}}{\sum_{j}^{n}X_{j}^{2}-n{\overset{¯}{X}}^{2}}$$

(3) $$\beta_{i}=\overset{¯}{RF_{i}}-LRT_{i}\cdot \overset{¯}{X}$$

(4) $$ERR_{i}=\frac{\sum_{j}^{n}\left|RF_{ij}-\left(LRT_{i}\cdot X_{j}+\beta_{i}\right)\right|}{n}$$

$\overset{¯}{X}$ is the mean of the number of treatments. $RF_{ij}$ denotes the features extracted from the *j*-th daily CBCT scan, $\overset{¯}{RF_{i}}$ is the mean of the N daily CBCT feature. Each feature of each patient can be characterized with a feature-specific $LRT_{i}$ and $\beta_{i}$.

Then, substitute the specific $LRT_{i}$ and $\beta_{i}$ into Equation (5) as the LRT feature (LRTF) of each feature for patients. Subsequent feature selection and modeling will be based on LRTF.

(5) $$LRTF_{i}=LRT_{i}+\beta_{i}$$

#### 2.1.4. Feature Reduction and Modeling

Feature selection was performed by univariate Mann–Whitney U-test between the responder and non-responder groups, with the retained features resulting in significant LRTFs with *p*-values less than 0.05 in the U-test. The U-test ensures that there is a difference in the features between the patients who responded to RT. For the LRTFs retained in the U-test, univariate logistic regression models were utilized for dimensionality reduction, keeping LRTFs that were correctly classified in the validation set. This step makes the retained features tend to have high metrics in the validation set. Final retained LRTFs are tested and modeled using a random forest (RF) algorithm to create a model that monitors and predicts early patient response during the radiation treatment delivery course. We used the predicted probability of responding patients as the RF score and the predicted probability comes from the results of the RF model; the RF model was performed using scikit-learn (version 0.24.2) [38].

We build a statistical weight (SW) model to interpret the RF results for clinical reference. The SW model was carried out by directly weighting the LRT and β obtained after fitting all features. The results of the univariate Mann–Whitney U-test reserved features to calculate the mean of the fitting features LRT and β; each LRT and β are weighted by univariate U-test *p*-values, weight formula (6).

(6) $$w=\frac{0.05-p}{0.05}$$

After obtaining the mean value of LRT and β and substituting them into Formula (5), we also obtain the LRTF. We calculated the accuracy for the two categories of LRTFs using a threshold of 0; a score greater than 0 suggests a potential good responder, while less than 0 suggests a poor responder patient.

For each patient, the LRT framework intervenes and start to work from the second fraction. For a treatment course of 28 fractions, 27 pairs of independent experiments, involving image features of the 1st fraction and kth fraction, were conducted to assess the treatment response of the patient. Leave-one-out cross-validation (LOOCV) for each experiment was performed to confirm the stability and potential of early treatment prediction of a therapeutic effect on the patient. Such experiments were performed on the image features based on the GTV region and the PTV region derived from CBCT scans, respectively.

The LRT framework was compared to radiomic models. Three radiomic models were created to predict post-RT treatment response: (1) radiomic model based on pre-treatment radiomic features; (2) during-treatment radiomic model based on 28 daily CBCT scans; (3) combined model using pre- and during-treatment radiomic features. In the radiomic models, the same imaging pre-processing, extraction, and reduction were used for training and prediction on the same patient cohort.

To evaluate model performance, Area Under Curve (AUC) and accuracy were utilized. We introduced a consistency index (CI) to evaluate the robustness of the LRT model. The CI is defined as whether it was correctly classified in the LOOCV experiments, regardless of whether the patient is included in the training data. The experiment with correct classification is recorded as 1, otherwise it is recorded as 0, and the mean of all experiments is calculated as the consistency of 6 patients.

### 2.2. Validation

We evaluated the proposed framework using a cohort of 30 patients (21 male and 9 female patients) diagnosed with LARC at stage II–IV. The average age (and standard deviation) of the patient cohort is 55.9 ± 11 (yr). The patient demographic information is summarized in Table 1. All patients underwent conventional neo-adjuvant fractionated radiation treatment with a prescription dose of 50.4 Gy (28 fractions) on a C-arm accelerator. The gross tumor volume (GTV) was delineated on the planning CT with the guidance of MRI or PET images. The clinical tumor volume (CTV) is the rectum and mesentery, including the perirectal, pre-sacral, and internal iliac nodes and the posterior portion of the obturator nodes. The CTV was expanded 1 cm into the posterior bladder (for bladder variation above vagina/prostate). The planning target volume (PTV) was generated using a 0.5–0.7 cm margin on the CTV. Prior to each treatment delivery, a daily kilovoltage CBCT image was acquired to ensure proper patient alignment, resulting in a total of 840 CBCT scans for the patient cohort. Four to eight weeks after the completion of CRT, all patients underwent total mesorectal excision surgery. Patients were separated into good-responder (GR) and non-GR groups according to the postoperative pathology report, MRI, or colonoscopy. The GR group (14 patients) consisted of patients with either complete clinical response (cCR) or partial response (PR), and the non-GR group consisted of patients with stable disease (SD) and progressive disease (PD).

## 3. Results

**Figure 2:**
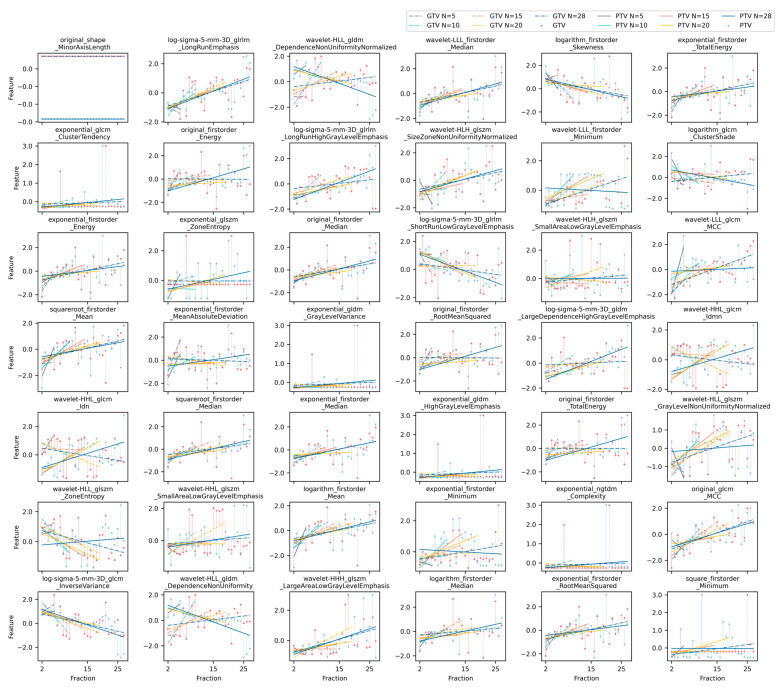
The least-squares fitting results for 42 preserved imaging features as a function of # of fractions for a representative patient; *x*-axis is # of fractions, and *y*-axis is the selected feature values derived from the GTV and PTV. The blue triangle points in the figure represent the feature values in the PTV of CBCT images during 28 treatments, the red circular points represent feature values of the GTV, the dotted line represents the straight line fitted by the features of the GTV area, and the solid line represents the straight line fitted by the features of the PTV. The distance between the point and the line represents the fitting error. The lines of five colors in the figure, DarkGray, MediumTurquoise, Orange, Gold, and Medium Blue, respectively, represent five groups of experiments with # of fractions of 5, 10, 15, 20, and 28.

Figure 2 shows the selected 42 features for a sample patient. Each subplot shows the fitting of each selected imaging feature when all N fractions of the CBCT scans, i.e., N = 5, 10, 15, 20, 28, were considered, respectively. Most of the features in the figure show an increasing or decreasing relationship in the overall trend of value transformation.

**Figure 3:**
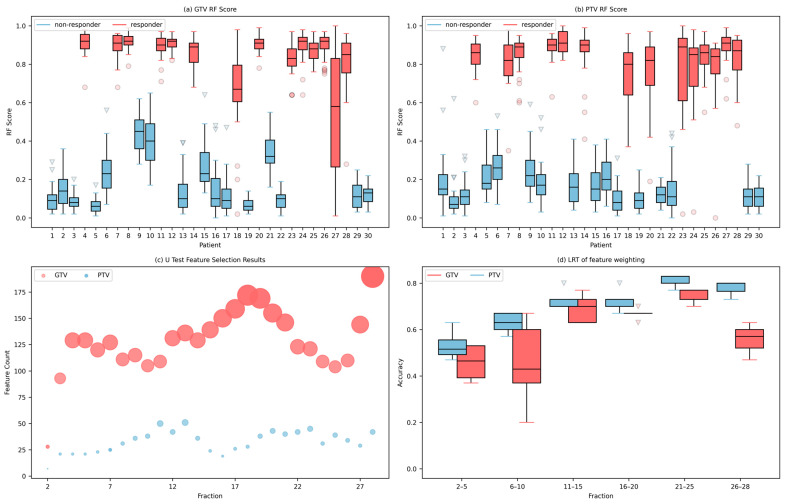
Box plots of patient RF scores using features derived from GTV (**a**) and PTV regions (**b**) for the cohort of LARC patients. The red boxes are responders, and the blue boxes are non-responder patients. (**c**) Number of features retained after the U-test. The red circles are the feature counts from the GTV, and the blue circles are the features derived from PTV. Larger size of circles means more features are retained. (**d**) Boxplot of model predictive accuracy using CBCT scans of previous n fractions. The red boxes are the GTV, and the blue boxes are the PTV.

Random forest model yielded AUCs of 0.98 (95% CI [0.97, 0.99]) and 0.9867 (95% CI [0.969, 1.0]), respectively, for GTV and PTV regions through LOOCV in 27 experiments. Figure 3a,b are the box plots of RF scores in 27 experiments for the GTV and PTV regions, respectively. The red box are responders, and the blue box are non-responders. Significant differences were observed in RF scores among responders and non-responders. Figure 3c visualizes the number of features retained after the U-test. Figure 3d illustrates the trend of the accuracy rate change with the increase in the number of treatments with every five treatments used as a group statistically. Before the 15th fraction, the accuracy increases with increasing fractions, after the 15th fraction, the predictive accuracies becomes more stable. Although the GTV regions preserve more features than the PTV regions, the overall accuracy of the GTV area is lower than that of the PTV area, as it can be seen from Figure 3d.

**Figure 4:**
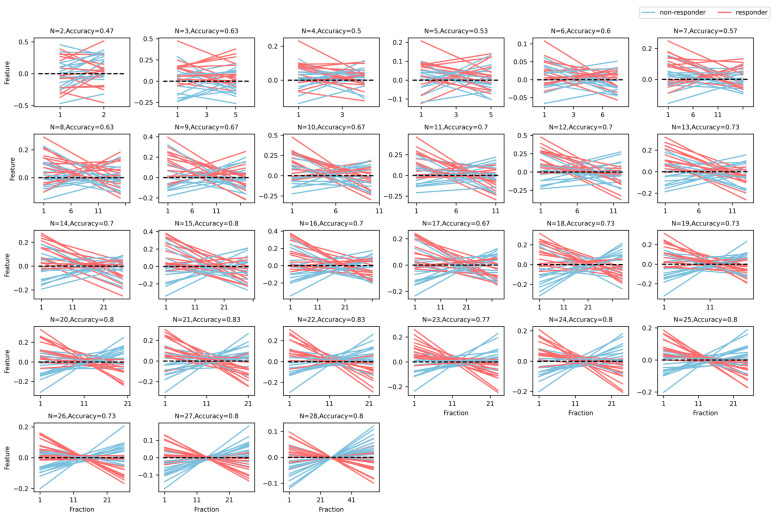
Trending patterns of the LRT derived from PTV features using SW model for all 30 patients. The red lines are the responders, the blue lines are the non-responder patients, with 0 as the threshold for the classification accuracy of the SW model.

Figure 4 shows the trending patterns of the LRT derived from PTV features using the SW model for all 30 patients, and the model results for N fractions are shown separately, where N = 2–28. The red lines denote trending plots for the responders, the blue lines are for non-responder patients, and the classification accuracy of the SW model uses 0 as the threshold. The classification model is directly weighted and summed based on the U-test feature screening results. The average accuracy of the GTV and PTV regions in 13 experiments before the first 15 treatments (N: 2–14) is 0.53 and 0.62, respectively. The average accuracy of six experiments with features from 15 to 20 treatments (N: 15–20) is 0.662 and 0.738, respectively, while the average accuracy of features from more than 20 treatments in eight experiments (N: 21–28) is 0.671 and 0.795, respectively.

**Figure 5:**
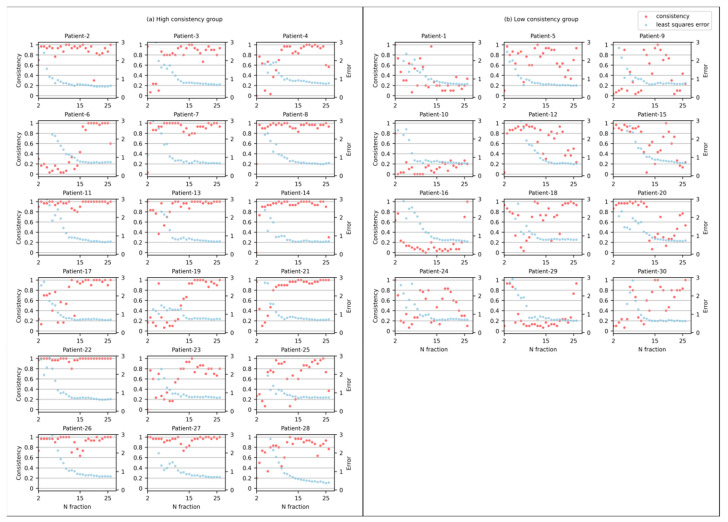
Consistency as a function of # fraction for (**a**) the high-consistency group, and (**b**) the low-consistency group. The red dots mean patient consistency (left *Y*-axis), the blue dots mean patient least-squares fit error (right *Y*-axis).

Figure 5 shows the consistency of the LOOCV experiments and the least-squares fit error mean of all features at Nth fractions, where N = 2–28, for each patient. To better interpret the results, the patients were divided into two groups based on the consistency mean after 15 fractions; the consistency means greater than 0.8 are defined as the high-consistency group, and the consistency means smaller than 0.8 are defined as the low-consistency group. For patients in the low-consistency group, a certain degree of overfitting was likely caused by the small sample size, especially for those patients with response scores of 1 and 2. Consistency analysis allows us to be more cautious about high indicators that appear in machine learning models. The group of patients with high consistency increased significantly after 15 factions, the mean consistency value increased from 0.71 to 0.92, and the least-squares fit error declined from 2.69 to 0.715, suggesting an improved classification ability when 15 or more factions are considered.

## 4. Discussion

Accurate and early prediction of patient response is critical for subsequent treatment management and adaptive planning. A longitudinal radiomic trend framework was proposed to systematically assess treatment response using CBCT in the work. This platform automates the process for the early assessment of patient response for routine clinical practice.

In the current trending analysis, we assumed that the image feature changes during the treatment course of LARC patients could be mapped to the final treatment effect through a linear relationship. Given the patients’ heterogeneity, the simple linear relationship may or may not be true for all patients. Despite being a simplified linear model, our model achieved great predictive accuracy and consistency for the cohort of LARC patients in the study. Specifically, accurate predictions could be achieved as early as the 15th fraction for the patient cohort (Figure 3d). We expect that the current platform can be utilized to access non-linear relationships between longitudinal imaging series and clinical endpoints for other disease sites. Artificial intelligence (AI)/deep learning (DL) is expected to be a good alternative for predicting non-linear feature transformation trends [39,40]. However, DL approaches generally require large data sets to train and evaluate the predictive models. Given the limited data size, overfitting and other issues are likely to occur. The proposed framework is designed to be flexible to incorporate both linear and non-linear trending models for outcome prediction for different cancer types.

In the comparative experiment of imaging analyses from the GTV area versus those from the PTV area, the predictive models created based on the GTV area were less predictive than the models created from the PTV area for trending analyses and radiomic experiments using either daily fractional CT solely or a combination of daily CBCT and pre-treatment CT. This can be understood in that the predictive models, when nodal area was considered, will better capture comprehensive underlying characteristics and therefore offer a better performance.

We evaluated robustness of the LRT by analyzing the consistency of 27 sets of LOOCV experiments for each patient. A higher consistency index demonstrates that the patient’s characteristics display more stability; caution should be exercised in evaluating the high accuracy achieved in machine learning models with patients who exhibit lower consistency. Compared to the machine learning models, we achieved the accuracy of 0.8 via the proposed SW models, which suggests that the LRT can separate the responders from the non-responders with decent performance. Analysis based on clinical data shows that the staging system and clinical features cannot differentiate between responders and non-responders.

Advantages of the proposed trending methods are more predictive than conventional ML-based delta-radiomic methods given the limited amount of data. In pre-treatment CT-based radiomic prediction models, the AUCs of the GTV and PTV radiomic feature sets were 0.43 and 0.62, respectively. In the CBCT-based radiomic prediction model, the AUCs ranged from 0.15 to 0.58 using the series of image features derived from the GTV region, and the AUCs ranged from 0.42 to 0.62 using the features derived from the PTV region. In the combined model, the AUCs ranged from 0.07 to 0.72 using the Image features from the GTV region, and the AUCs ranged from 0.27 to 0.65 using the features from the PTV region. The ML-based radiomic prediction models suggested serious overfitting due to the relatively small number of the data sample. The LRT may be considered similarly to project high-dimensional spatial features to low-dimensional space, which makes complex feature variables simple and useful. The proposed LRT framework, focusing on trending analysis throughout the entire course, yields more effective and robust results and maintains a good generalization ability even for relatively small data sets.

The proposed automated framework LRT platform aims to streamline the clinical utilization of longitudinal quantitative images for treatment response prediction. The platform achieved good performance for the cohort of LARC patients. The target region is currently obtained through registration from the planning GTV/PTV to the CBCT scans during the course of radiation treatment, which may or may not capture actual target volume changes. Yamashita et al. reported a tendency of rectal volume reduction during weekly CBCT through neoadjuvant concurrent chemoradiation therapy for rectal cancer [41]. Zumre et al. demonstrated that the mesorectum moved 20 mm laterally and in the anterior/posterior direction and up to 10 mm in the inferior/superior direction [42]. Although certain target volume changes are eliminated through image registration, accurate segmentation is important for accurate predictions. Furthermore, it is known that CBCT images are subject to poor quality and artifacts, which may be a big challenge for stable radiomic features. Our future work will focus on improvement of CBCT image quality to achieve high performance of automated segmentation for the targets. Lastly, the proposed LRT can alleviate the instability brought by image quality to some extent, which mayfurther reduce fitting error of the LRT and increase its confidence level.

## 5. Conclusions

We developed a framework that integrates image registration, quantitative feature extraction, and longitudinal imaging trending analysis to predict post-treatment clinical outcomes. The effectiveness of the framework was validated for a retrospective cohort of LARC patients with superior performance, allowing for systematic monitoring and the early assessment of patient response as early as the 15th fraction during the RT treatment course for potential adaptive management.

## Figures and Tables

**Table 1:** Patient demographic information.

Clinical Characteristics	Good Responder (GR)	Poor Responder (PR)
# of cases	14	16
Gender, n (%)		
Male	9 (64.3%)	12 (75%)
Female	5 (35.7%)	4 (25%)
Average age (standard deviation) (yr)	52.3 ± 11.7	59.2 ± 9.3
Stage, n (%)		
II	3 (21.4%)	4 (25%)
III	8 (57.2%)	9 (56.3%)
IV	3 (21.4%)	1 (6.3%)
Unknown		2 (12.4%)
Response scores, n (%)		
0	7 (50%)	
1	7 (50%)	
2		11 (68.8%)
3		5 (31.2%)

## Data Availability

The data presented in this study are available upon request from the corresponding author.
